# Effects of phospholipase C inhibition on the regulation of membrane lipid metabolism in maize leaves

**DOI:** 10.3389/fpls.2025.1547477

**Published:** 2025-04-01

**Authors:** Yulei Wei, Zhicheng Cai, Zhiyuan Che, Xu Guo, Shengnan Ge, Xinyang Che, Jie Deng, Haiyang Zhang, Lin He, Jingyu Xu

**Affiliations:** Key Laboratory of Modern Agricultural Cultivation and Crop Germplasm Improvement of Heilongjiang Province, College of Agriculture, Heilongjiang Bayi Agricultural University, Daqing, China

**Keywords:** maize (*Zea mays* L.), phospholipase C, neomycin sulfate, transcriptome, lipidome

## Abstract

**Introduction:**

Phospholipase C (PLC) is an enzyme that catalyzes the hydrolysis of glycerophospholipids and can be classified as phospholipase-specific PLC (PI-PLC) and non-specific PLC (NPC) depending on its specific substrate.

**Methods:**

In this study, neomycin sulfate (NS, 100 mM) was used to inhibit the activity of phospholipase C in maize seedlings, and the effect of phospholipase C on lipid metabolism was investigated by combined analysis of transcriptome and lipidome.

**Results:**

Lipidomic analysis showed that when PLC was inhibited, the content of phospholipids showed more than 10% increase due to the elevated accumulation of PC and PE. At the same time, transcriptomic data suggested an activation of the de novo biosynthesis of PC and PE from choline and ethanolamine via upregulated CCT and PECT, respectively. Thus, the inhibition of phospholipid hydrolysis and the enhancement of de novo synthesis together contribute to the increase in the total phospholipids. Glycolipids account for around 60% of the total lipids in leaves. Under NS treatment, MGDG decreased significantly, while DGDG increased.

**Discussion:**

Our results indicate that NS treatment may cause damage to photosynthetic membrane lipids, whereas the increase in bilayer lipid DGDG may provide a kind of protection to maintain the stability of chloroplasts. These findings suggest that phospholipase C plays a key role in plasma membrane metabolism.

## 1 Introduction

Plants contain a variety of lipids, such as phospholipids and glycolipids, which are major components of biological membranes (Bahammou et al., 2024; Seth et al., 2024), key signaling molecules in response to plant development and a variety of environmental stresses (Pokotylo et al., 2013), and are important for maintaining both cellular and organelle integrity (Seth et al., 2024). Phospholipids can be degraded by phospholipases into a variety of products such as diacylglycerol (DAG), phosphatidic acid (PA), free fatty acids (FFA) and lysophospholipids (LPLs) (Zhu et al., 2020). In plants, phospholipases are categorized into three groups, namely Phospholipase A (PLA), Phospholipase C (PLC), and Phospholipase D (PLD) (Hong et al., 2016; Takáč et al., 2019). Among them, phospholipase C is considered as an important lipid hydrolase in plants and animals with profound effects on membrane lipid remodeling and intracellular signaling (Meldrum et al., 1991);

Based on different substrate affinities and cellular functions, plant phospholipase C can be categorized into phosphatidylinositol-specific PLCs (PI-PLCs) and phosphatidylcholine PLCs (PC-PLCs) (Kocourková et al., 2011; Pokotylo et al., 2014). PI-PLC hydrolyzes phosphatidylinositol to produce inositol 1,4,5-trisphosphate (IP3) and diacyl glycerol (DAG) (Berridge, 1987; Meldrum et al., 1991), which can be phosphorylated and converted to PA by DAG kinase (DGK) (Wang et al., 2006). Unlike PI-PLC, PC-PLC preferentially hydrolyzes PC but can also act on other lipids, such as phosphatidylethanolamine (PE) and phosphatidylserine (PS), and is therefore also known as non-specific phospholipases C (NPC) (Kocourková et al., 2011; Pokotylo et al., 2014).

The process of PC synthesis occurs on the endoplasmic reticulum (ER) and is catalyzed by amino alcohol phosphotransferase (AAPT), which transfers P-choline from CDP-choline to DAG (Botella et al., 2017; Nakamura, 2021). This process of DAG is mainly derived from the Kennedy pathway, whereby glycerol 3-phosphate (G-3-P) is transferred from CDP-choline to DAG *via* glycerol 3-phosphate acyltransferase (GPAT) and lysophosphatidic acid acyltransferase (LPAT) (Botella et al., 2017; Nakamura, 2021). Hydrolysis of PC involves multiple metabolic pathways (Botella et al., 2017), PC can be hydrolyzed to PA and choline *via* PLD, and to DAG and P-choline *via* PLC. PA and DAG derived from PC are substrates for the synthesis of most non- plastids phospholipids and glycolipids (Boudière et al., 2014). PA is a key component of the plasma membrane, playing critical roles in protecting cellular integrity, regulating nutrient exchange, and mediating signal perception (Grison et al., 2015; Mamode Cassim et al., 2019; Zhou et al., 2024). Additionally, PA serves as a biosynthetic intermediate for membrane lipids such as PG and PI, contributing to the formation of chloroplast and mitochondrial membrane systems (Yao et al., 2024). Previous studies suggested that, in 18:3 plant maize, the biosynthesis of MGDG and DGDG depends on phospholipase PLD and PLC mediated hydrolysis of phospholipids to provide the DAG precursors (Gu et al., 2017; Zhao et al., 2021).

In previous studies in our laboratory, it was found that when phospholipase C activity was inhibited, maize seedlings exhibited stunted growth and development and decreased photosynthesis (Wei et al., 2022). To further analyze, the effect of phospholipase C on phospholipid metabolism, we used a combined strategy of lipidomics and transcriptomics to investigate the lipidomic changes and transcriptional regulation of maize seedling leaves under conditions of inhibition of phospholipase C activity, in order to clarify the role of phospholipase C in the metabolism of leaf lipids.

## 2 Materials and methods

### 2.1 Plant growth, handling and sampling

The maize inbred He344 was used in this study. Maize seeds of the same size were selected and disinfected with 10% NaClO for 30 min. After repeated washing with dis-tilled water, the seeds were placed in an incubator at 25°C for dark germination. After germination, maize seedlings were grown in a growth chamber with 1/2 Hoagland’s nutrient solution (pH=5.5). The temperature of the growth chamber was set at (25 ± 2) ℃/(20 ± 2) ℃, with a photoperiod of 16 h/8 h (light/dark) and 60%-80% humidity. A solution of 1/2 Hoagland’s nutrient in two-week-old maize seedlings was added with Neomycin Sulphate (NS) to give a final concentration of 100 mM, and the inhibitory nutrient solution was changed every three days to inhibit phospholipase C. Maize leaf samples were collected at 1, 2, 3, 5, and 7 d after the NS inhibition treatment. The collected samples were wrapped in Tin Foil and quickly frozen in liquid nitrogen and stored at -80°C.

### 2.2 Membrane lipid extraction and analysis

The method was modified according to a previous report (Narayanan et al., 2016). 3 ml isopropanol (0.01%BHT) was added to a 50 ml glass tube and the glass tube was placed in the nitrogen blowing instrument and preheated to 75℃. About 200 mg maize root samples were rapidly added to the preheated glass tube and kept at 75℃ for 15 min. Distilled water (0.6ml) and chloroform (1.5ml) were added to the tube, vortexed and shaken for 1 h in a shaking table, and then the extract was transferred to a new glass tube. Next, 4 ml chloroform: methanol (2:1) mixture was added to the glass tube, vortexed and shaken for 30 min on a shaking table. The extraction procedure was repeated 3-4 times, and then the mixed liquids were compounded and washed with KCl (1 ml). The upper liquid was discarded, and the remaining liquid was blown to full evaporation with nitrogen blowing instrument, and then stored at -20℃. Lipids were analyzed by Electrospray Ionization-mass Spectrometry (ESI-MS/MS), which was accomplished at Kansas Lipidomics Research Center (KLRC, United States). Different lipids were identified based on the mass of intact ions (m/z) as well as the mass of individual fragment ions formed in the mass spectrometer, with an accuracy of more than 99% by excluding missing values. The signals of the sample peaks were compared to the signals of the peaks of a known amount of the internal standard and the data were reported so that the signals could be expressed as 1 = the same as the 1 nmol internal standard. The normalized signal was obtained by normalizing all lipid signals and then obtaining the normalized signal/(tissue index of the sample), which is the molar percentage (%) of the different lipids.

### 2.3 Statistical analysis of lipid content

All statistical analyses were performed using SPSS Statistics 24.0 (SPSS Inc.) and the significance level of the data was calculated by Student’s t-test. *P<0.05 and **P<0.01 indicate different levels of significance. Histogram plotting is done using ggplot2 package, tidyr package, dplyr package, ggsignif package and ggsci package in R. PCA plotting is done using ggrepel package and correlation heatmap is done using pacman package and vegan package.

### 2.4 Lipid dendrogram clustering analysis

Lipid clustering analysis was performed with reference to the method used by Narayanan (Narayanan et al., 2016) and lipid data for each genotype were uploaded to CLUSTER 3.0 (open-source software, Human Genome Center, The Institute of Medical Science, The University of Tokyo, Tokyo Japan) (Eisen et al., 1998) for analyzing lipid clusters. CLUSTER 3.0 used a single-chain hierarchical algorithm based on Spearman’s correlation coefficient ρ to generate lipid clusters for each genotype. The gtr files and cdt files obtained from CLUSTER 3.0 were converted to NEWICK format files using ape package in R language. The obtained NEWICK files were exported to MEGA7 to generate dendrograms and based on this dendrogram color modification was done.

### 2.5 Transcriptomic data analysis

DEGs (differentially expressed genes) were analyzed using a false discovery rate (FDR) of less than 0.01 and |log2 FC| ≥ 1. The DEGs were annotated to the Kyoto Encyclopedia of Genes and Genomes (KEGG) (https://www.genome.jp/kegg/) database and Gene Ontology (GO) (www.geneontology.org) annotation database, respectively. Volcano mapping was performed using the tidyverse package and ggVolcano in R. UpSet mapping was performed using the UpSetR package, and heatmaps of differential gene clustering were performed using the ClusterGVis package, the ComplexHeatmap package, the clusterProfiler package, and the cols4all package.

## 3 Results

### 3.1 The effects of phospholipase C inhibition on the total glycerolipids in maize seedling leaves

In order to investigate the role of phospholipase C in the lipid metabolism process, its inhibitor-neomycin sulfate (NS) was applied on maize seedlings, after which the glycerolipid in the leaves of maize seedlings were extracted and analyzed. We detected a total of 12 lipids, including two glycolipids (MGDG, DGDG), one neutral lipid (DAG), six phospholipids (PC, PE, PA, PI, PS, and PG), and three lysophosphatidylcholines (LPC, LPE, and LPG).

**Figure 1:**
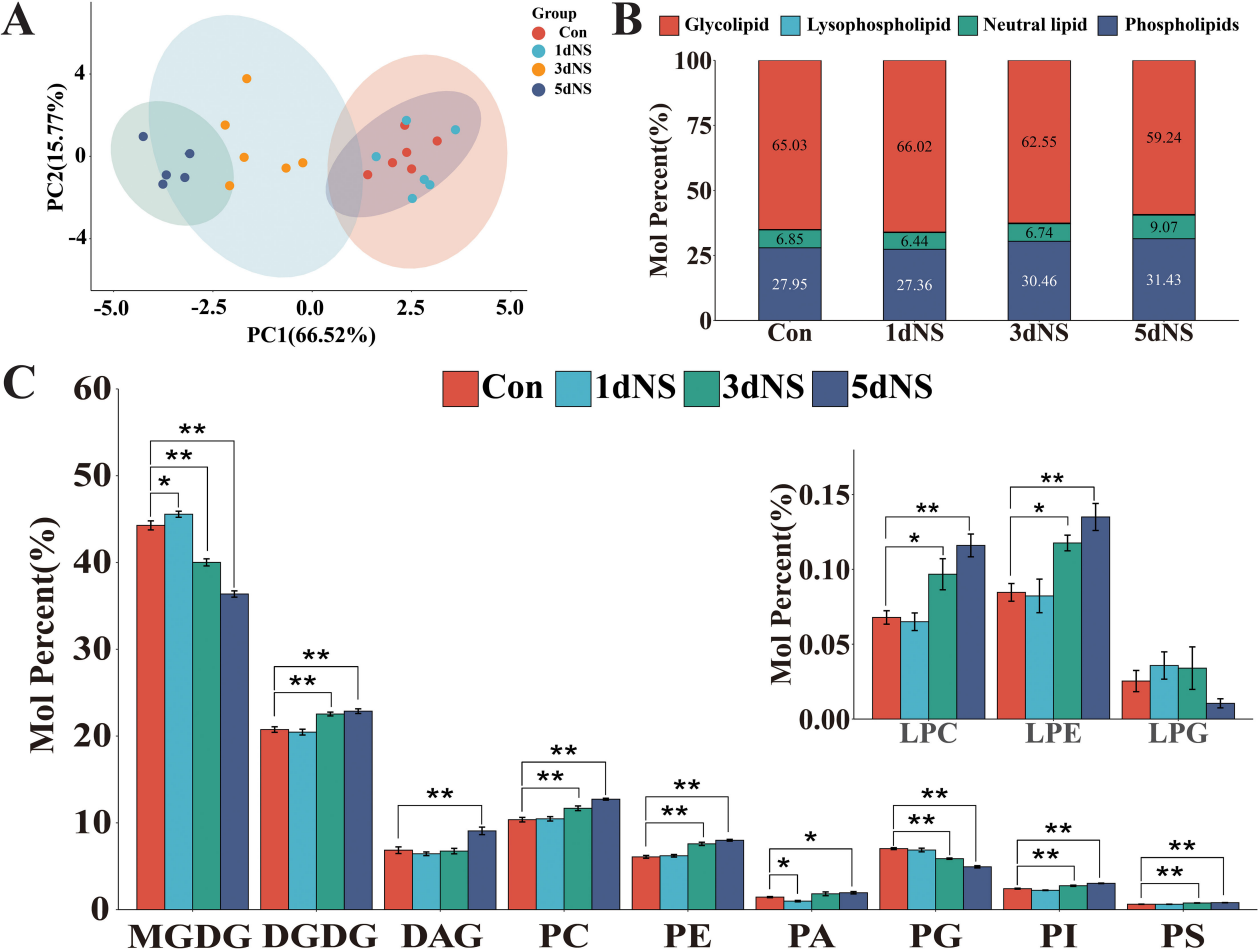
Changes in glycerolipids in maize seedling leaves under neomycin sulfate (NS) inhibition. **(A)** PCA analysis of glycerol lipids; **(B)** The proportion of different glycerolipids; **(C)** Changes in glycerolipid content; Values (mol %) are the mean 6 ± standard deviation (SD) (n = 6) “*” indicates that the value is significantly different from the control. (P<0.05). "**" means that the value is extremely different from the control. (P<0.05).

As shown in **Figure 1**, the PCA analysis of lipids (**Figure 1A**) showed different degrees of aggregation of lipid content with the increase of the inhibition time, which indicated that PLC was involved in the lipid remodeling process. In maize leaves, glycolipids constituted the majority of the lipids, about 60% of the total lipids, followed by phospholipids, which accounted for about 30% of the total lipids (**Figure 1B**). The molar percentage of glycolipids showed a decreasing tendency with the extension of processing time, whereas an increase in phospholipids and neutral lipids was observed (**Figure 1B**). Further analysis revealed that among the glycolipids, the molar percentage of MGDG was first increased and then decreased, and the molar percentage of DGDG decreased and then increased. PC, PE, and PG accounted for the major components in phospholipids. PG content was decreasing as the NS treatment extended. Interestingly, the molar percentage of the neutral lipid DAG was decreased at the beginning and then increased under the NS treatment (**Figure 1C**). However, all of these changes in lipid content are only minor changes. These results suggest that phospholipase C is involved in lipid regulation and remodeling processes.

### 3.2 The effects of phospholipase C inhibition on molecular species of phospholipids

**Figure 2:**
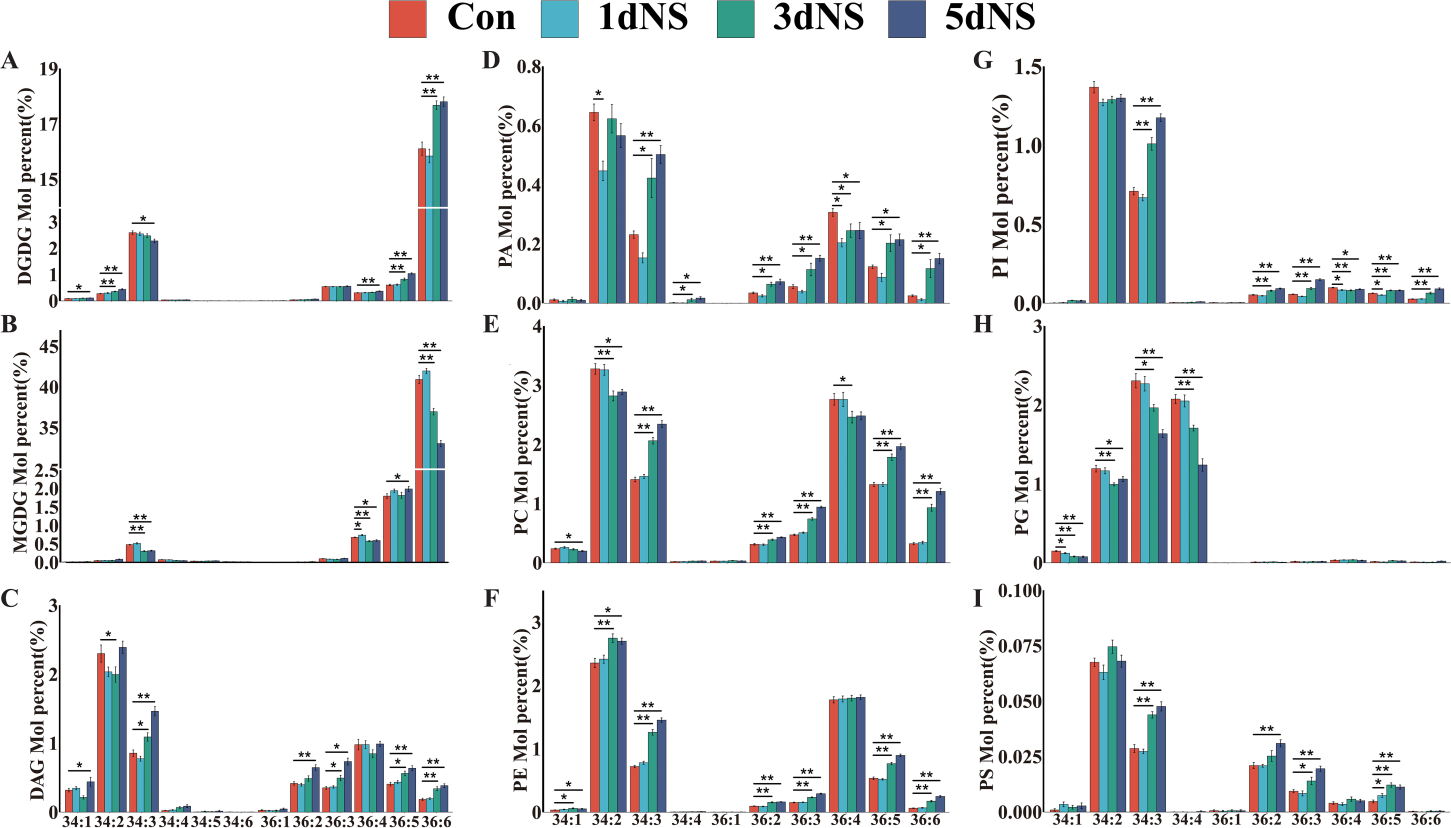
Changes in the molecular species of major glycerolipids in maize seedling leaves under neomycin sulfate (NS) inhibition. **(A)** Percentage change in the number of moles of the DGDG molecular species; **(B)** Percentage change in the number of moles of the MGDG molecular species; **(C)** Percentage change in the number of moles of the DAG molecular species; **(D)** Percentage change in the number of moles of the PA molecular species; **(E)** Percentage change in the number of moles of the PC molecular species; **(F)** Percentage change in the number of moles of the PE molecular species; **(G)** Percentage change in the number of moles of the PI molecular species number; **(H)** Percentage change in moles of PG molecular species; **(I)** Percentage change in moles of PG molecular species.Values (mol %) are mean 6± standard deviation (SD) (n = 6). “*” indicates that the value is significantly different from the control; "**" means that the value is extremely different from the control (P<0.05).

The molecular species of all glycerolipids were analyzed, and was presented as Cnn:n (total number of carbon atoms: total number of double bonds). The profiles of glycolipid MGDG and DGDG indicated that maize is a typical 18:3 plant, because the molecular species were dominated by C36 (**Figure 2**). During the process of 100 mM NS treatment, the level of C36:6 MDGD first increased and then decreased, reaching a minimum of 33.05 at 5dNS, a decrease of 19.23% compared to the control group. However, the level of C36:6 DGDG first decreased and then increased, reaching a maximum of 19.58 at 5dNS, which was an increase of 13.28% compared to the control. Neutral lipids DAG are mainly composed of C34 and C36, among which C34:2 and C36:4 accounted for a relatively high proportion. Compared with the control group, C34:2 DAG increased by 3.92% on the 5dNS. PC and PE are higher in the molecular species C34:2 and C36:4. Compared to the control group, C34:2 PC decreased by 11.85% and C36:4 decreased by 10.11% on 5dNS, while C34:2 in PE increased by 14.64%. C36:2 is the main molecular species of PA. Compared to the control, C36:2 in PA first decreased and then increased, rising 35% on 5dNS. In PI and PS, the proportion of C34:3 and C34:2 was relatively high. In PG, the proportion of C34:4 and C34:3 was relatively high. On the other hand, lysophospholipids were dominated by C16 and C18. These results indicate that in maize leaves, glycolipids were dominated by C36 molecular species, and neutral lipids and phospholipids were dominated by C34 and C36 molecular species.etc.

### 3.3 Clustering analysis on glycerolipids

**Figure 3:**
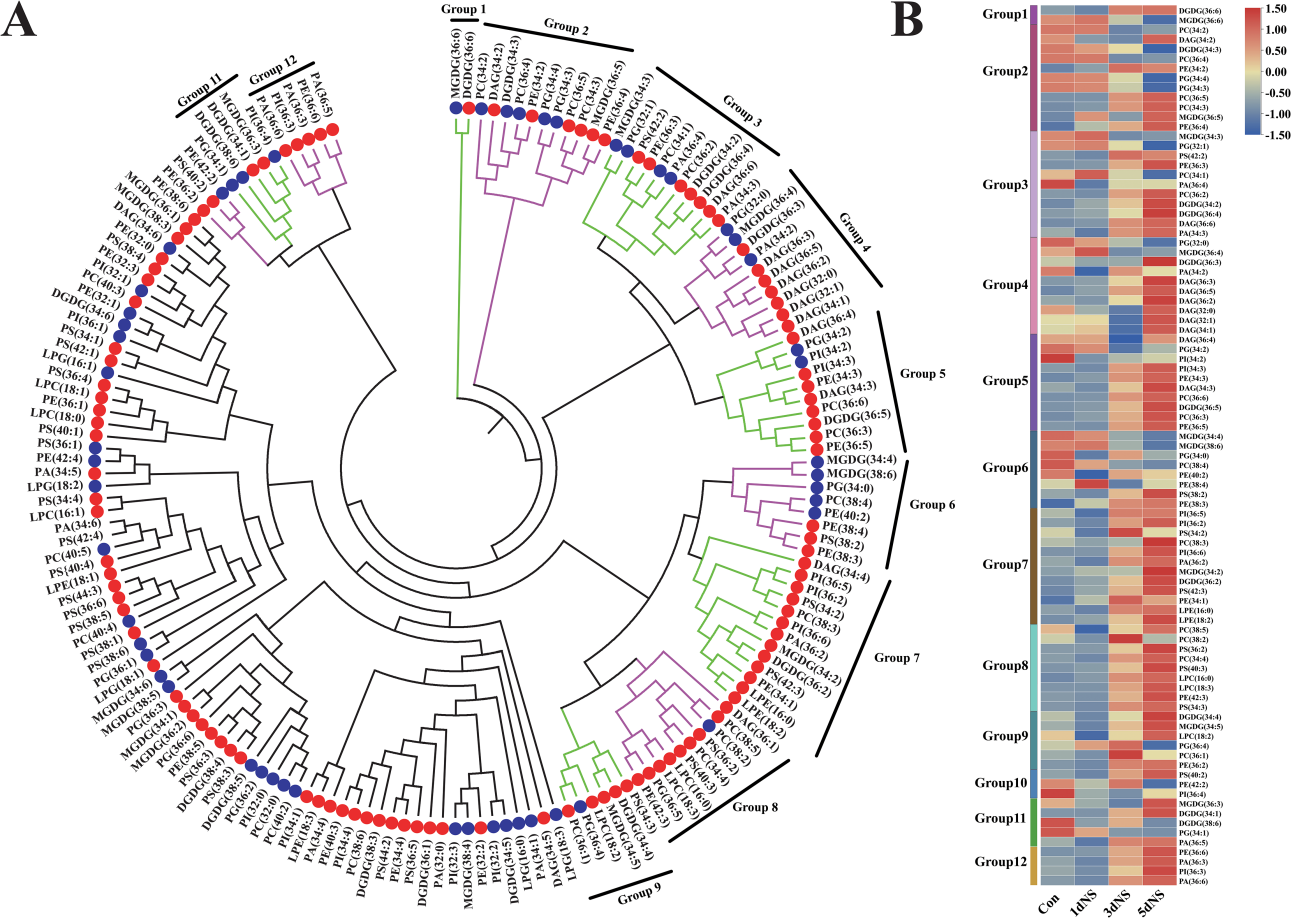
Correlation analysis of lipid molecular species under neomycin sulfate inhibition. **(A)** Genotypic lipid dendrogram of lipid molecular species. Green and purple bars on the dendrogram indicate the 12 clusters with ρ≥ 0.80. Coexisting lipid clusters consisting of whole clusters, partial clusters, or combinations of two neighboring clusters are indicated on the dendrogram. Circles on the dendrogram indicate the directionality of significant differences in 5dNS lipid content under inhibition; red circles indicate lipids with increased content and blue circles indicate lipids with decreased content. **(B)** Heatmap of the 12 clusters of lipids.

To investigate the correlation among different glycerolipid species, the Spearman’s correlation coefficient (ρ) was calculated by CLUSTER 3.0. The Spearman’s correlation coefficient ranges from -1 (perfect negative correlation, indicated in blue) to 1 (perfect positive correlation, indicated in red). As shown in **Supplementary Figure S1**, PC had a high positive correlation with PE (ρ = 0.90). Subsequently, each lipid species was matched to its most highly correlated analyte, and dendrograms of lipid species under treatment of NS inhibition were plotted (**Figure 3A**). These dendrograms contained different lipid clusters in which each lipid was correlated with at least one other lipid in the cluster with ρ ≥ 0.80. The dendrograms were divided into 12 clusters, indicated by green and purple bars on the dendrograms, and the circles on the dendrograms represented the change in the content of each lipid under 5dNS treatment compared with that of the control. The kinetic changes of individual lipids in each of the groups were presented as heat maps in **Figure 3B**, and most of the lipids showed varying degrees of accumulation in response to 100mM NS treatment. Interestingly, in group1, only MGDG (36:6) and DGDG (36:6) were clustered and showed a significant negative correlation, suggesting an interconversion between MGDG (36:6) and DGDG (36:6).

### 3.4 Transcriptomic analysis of maize seedling leaves under phospholipase C inhibition

**Figure 4:**
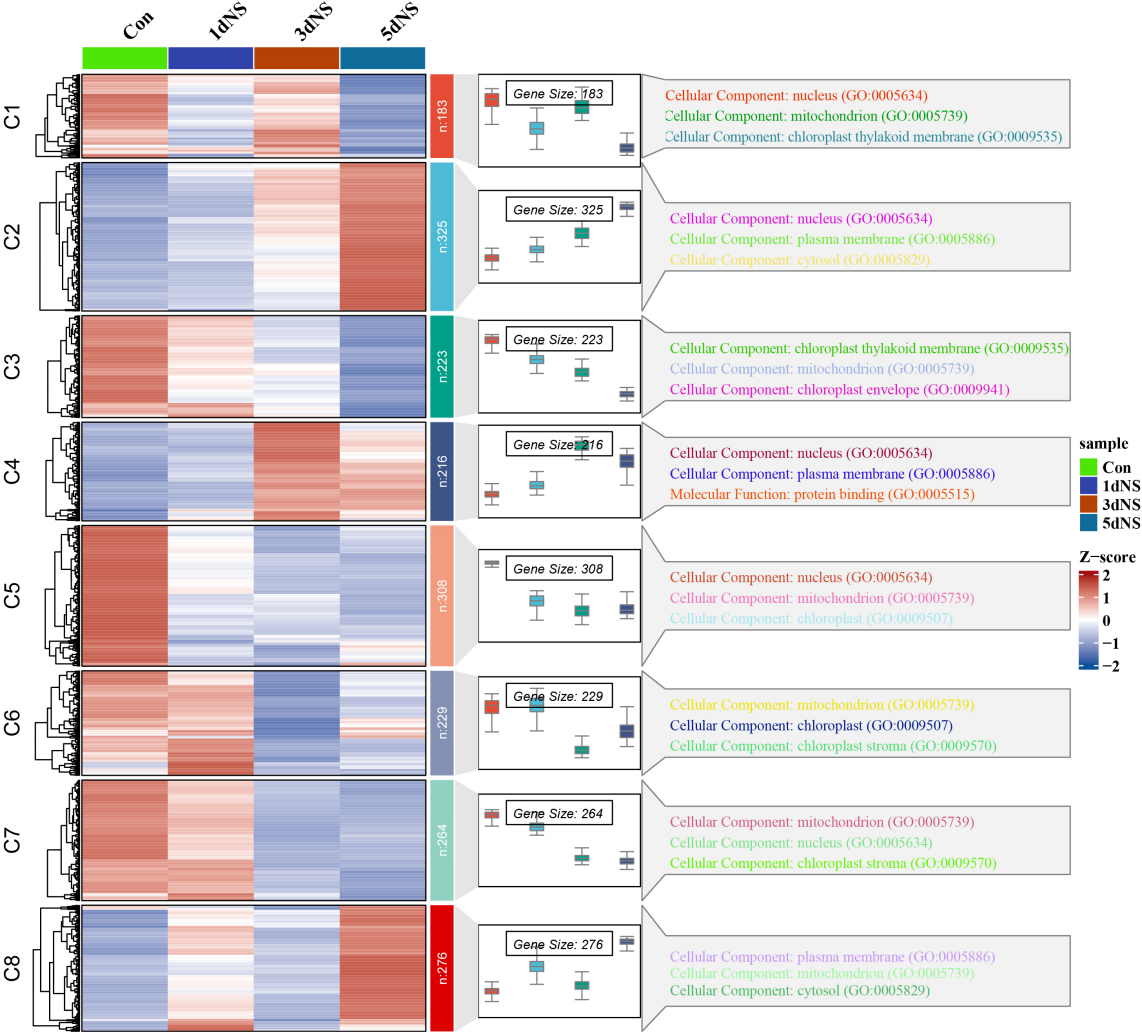
Temporal expression patterns and GO annotation analysis of DEGs under NS inhibition. The heatmap on the left indicates the expression of DEGs under different inhibition times; the box-and-line plot in the middle indicates the expression range of DEGs and the expression trend; and the GO annotation analysis of DEGs in different subgroups on the right.

In order to investigate the transcription of genes in response to phospholipase C inhibition, RNA-Seq analysis of RNA from leaves of maize seedling under 100mM NS treatment was conducted. The differentially expressed genes (DEGs) were screened with |log2FC|≥1 as the screening criteria. The gene expression of samples without NS treatment applied on day 0 was used as control (Con). The number of DEGs gradually increased with the increase of NS treatment time, and the highest number of DEGs was observed at 5 d after NS treatment (5dNS) (**Supplementary Figure S2A-C**. A total of 1726 DEGs were screened by Upset analysis in the “5dNS vs Con” comparison group, of which 689 were up-regulated and 1037 were down-regulated (**Supplementary Figure S2D**). The screened DEGs were analyzed by “spatio-temporal” expression clustering using the ClusterGVis package in R (**Figure 4**). The results showed that the screened DEGs could be categorized into 8 groups, i.e., groups C1-C8. Meanwhile, in order to further analyze the DEGs in each group, we associated to the Gene Ontology (GO) database and made GO annotations for the DEGs in each group. “Spatio-temporal” expression clustering analysis usually focuses on subgroups with more regular expression trends, and in this study we focused on C2 and C3. Among them, a total of 325 DEGs were clustered into group C2, which were upregulated with increasing time of NS treatment; group C3 was clustered with 223 DEGs, which were downregulated with increasing time of NS treatment. DEGs in groups C2 and C3 were mainly annotated in the “Cellular Component” category, which were further annotated in the “nucleus”, “chloroplast thylakoid membrane”, “chloroplast envelope”, “mitochondria and chloroplast stroma” GO subdivisions, indicating that when phospholipase C activity is inhibited, the expression of genes related to chloroplasts is disturbed, thereby destabilizing the chloroplast membrane.

### 3.5 Analysis of KEGG pathway enrichment of DEGs

**Figure 5:**
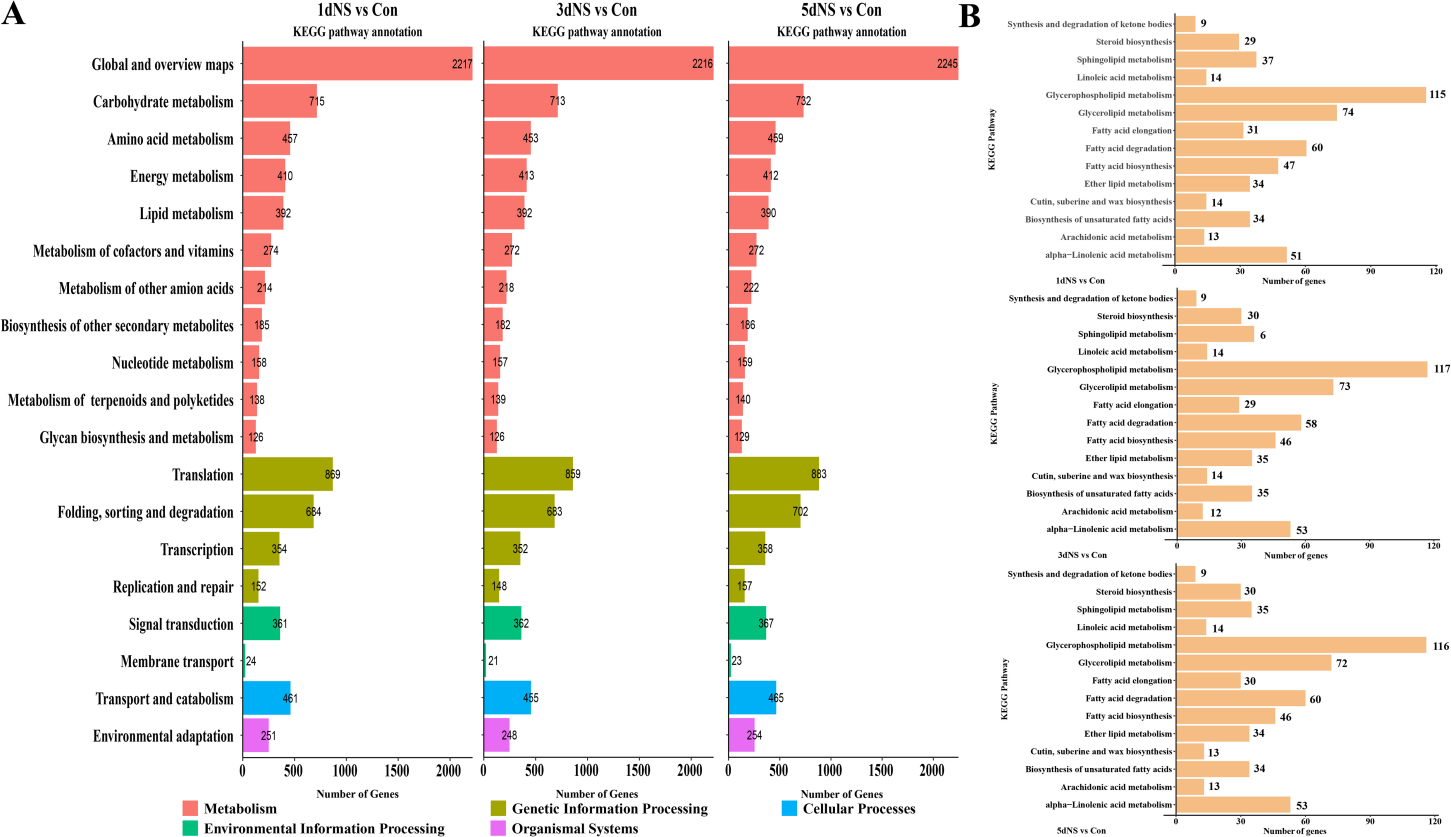
KEGG pathway annotation analysis of DEGs under inhibition of phospholipase C activity. **(A)** Annotation of DEGs to KEGG secondary pathway enrichment analysis in different treatment periods; **(B)** Annotation of lipid-related genes to KEGG tertiary pathway enrichment analysis in different treatment periods.

To explore the most significant biological processes in which DEGs are enriched, KEGG pathway annotation was conducted. The DEGs were mainly annotated into 19 KEGG categories based on their putative functions (**Figure 5A**), and more than half categories (11 out of 19) related to metabolic processes. The most representative metabolic categories were “Global and overview maps”, which has 2245 DEGs in ‘5dNS vs Con’, followed by “Carbohydrate metabolism”, “Amino acid metabolism”, “Energy metabolism”, and “Lipid metabolism”, as well as other secondary metabolic biosynthesis. Next, pathway enrichment analysis was performed on DEGs in the “lipid metabolism” categories, and a total of 14 pathways were annotated (**Figure 5B**). A large number of lipid-related genes were enriched in “Glycerolipid metabolism” and “Glycerophospholipid metabolism” pathways. With 115 and 74 DEGs enriched in ‘1dNS vs Con’ and 117 and 73 DEGs enriched in ‘3dNS vs Con’, respectively, and 116 and 72 DEGs enriched in ‘5dNS vs Con’, respectively(**Figure 5B**). These findings suggest that when PLC is inhibited, it has a significant impact on the metabolic pathways glycerolipid metabolism.

### 3.6 Combined transcriptomic and lipidomic analysis of glycerolipid metabolism pathways

**Figure 6:**
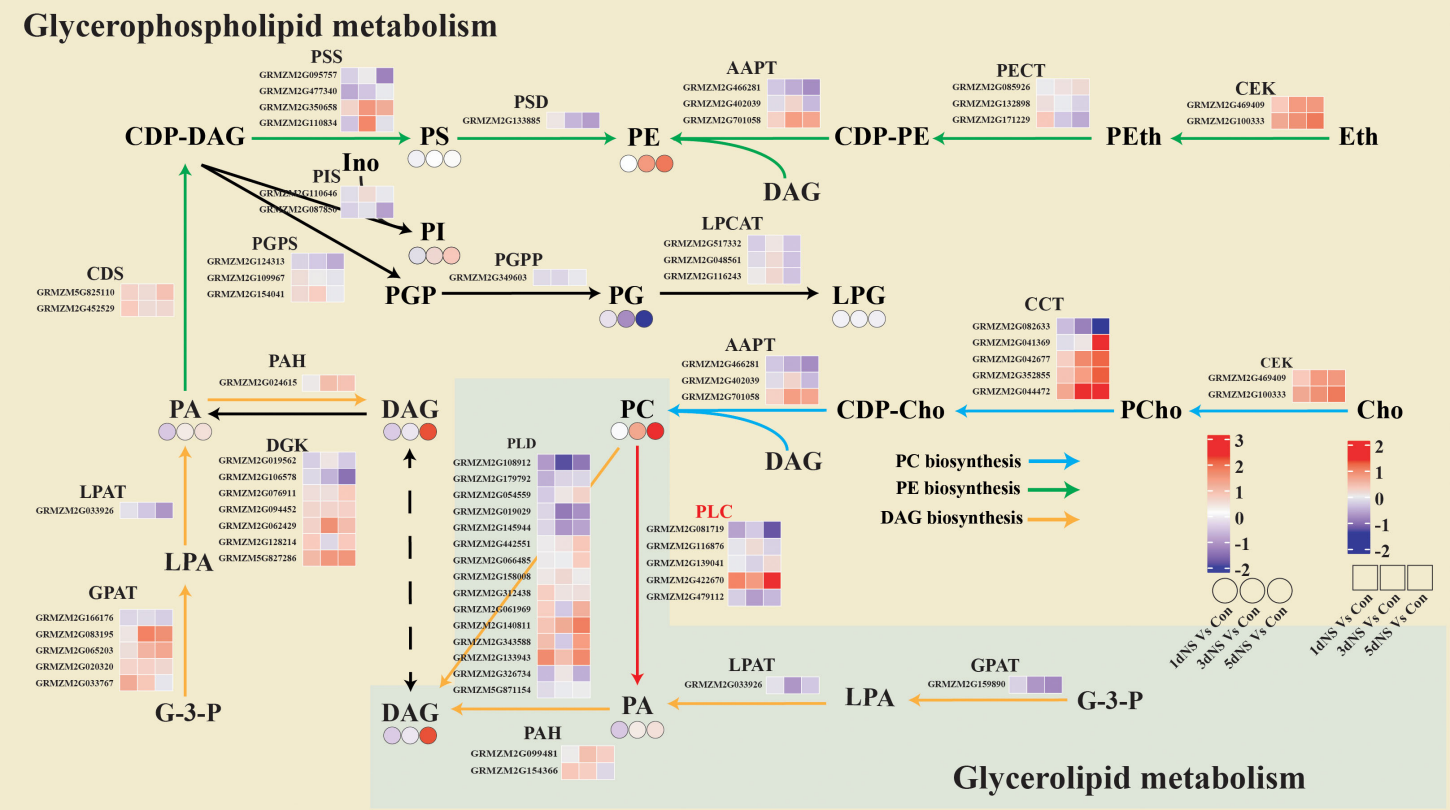
Combined transcriptomic and lipidomic analysis of glycerophospholipid metabolism and glycerolipid metabolism. The yellow background represents the “glycerophospholipid me-tabolism” pathway and the green background represents the “glycerolipid metabolism” pathway. Blue arrows represent PC biosynthesis pathway, green arrows represent PE biosynthesis pathway, yellow arrows represent DAG biosynthesis pathway, and blue arrows represent. The small squares represent heatmaps of differential gene expression for 1dNS vs Con, 3dNS vs Con and 5dNS vs Con, and the small circles represent heatmaps of changes in lipid content for 1dNS vs Con, 3dNS vs Con and 5dNS vs Con.

To better elucidate the regulation of glycerolipid metabolism, lipidomics and transcriptomics data were jointly analyzed and a metabolic regulatory network showing differential genes and differential metabolites was constructed (**Figure 6**). *De novo* synthesis of glycerolipids in “Glycerophospholipid metabolism” (ko00564) and “Glycerolipid metabolism” (ko00561) is achieved by glycerol-3-phosphate (G-3-P) acylation catalyzed by a series of acyltransferases, and the intermediate diacylglycerol (DAG) is also synthesized as a precursor of glycerophospholipids. The first step of the acylation reaction of G-3-P is catalyzed by GPAT. Six GPAT encoding unigenes were identified in the transcriptomic data, five GPAT encoding unigenes was involved in “Glycerophospholipid metabolism”, 4 of which were up-regulated under NS treatment, and one GPAT encoding unigene found in “Glycerolipid metabolism”, was down-regulated under NS treatment. Three unigenes encoding PAH, which converts PA to DAG, including one in the endoplasmic reticulum and two in the chloroplast, were also significantly up-regulated (**Figure 6**)

The *de novo* synthesis of the major phospholipids PC and PE is initiated by the activation of choline and ethanolamine and the condensation with DAG, respectively. As can be seen on **Figure 6**, two unigenes encoding the kinase CEK involved in choline and ethanolamine activation were significantly up-regulated at different time points under NS treatment. Multiple AAPT encoding genes that catalyze the reaction of CDP-Cho (CDP-Etn) with DAG to produce PC (PE) were also significantly up-regulated. The induced expression of these genes involved in the *de novo* phospholipid synthesis pathway may be directly related to the increased level of PC and PE under NS treatment.

### 3.7 Combined transcriptomic and lipidomic analysis of lipid molecular species

**Figure 7:**
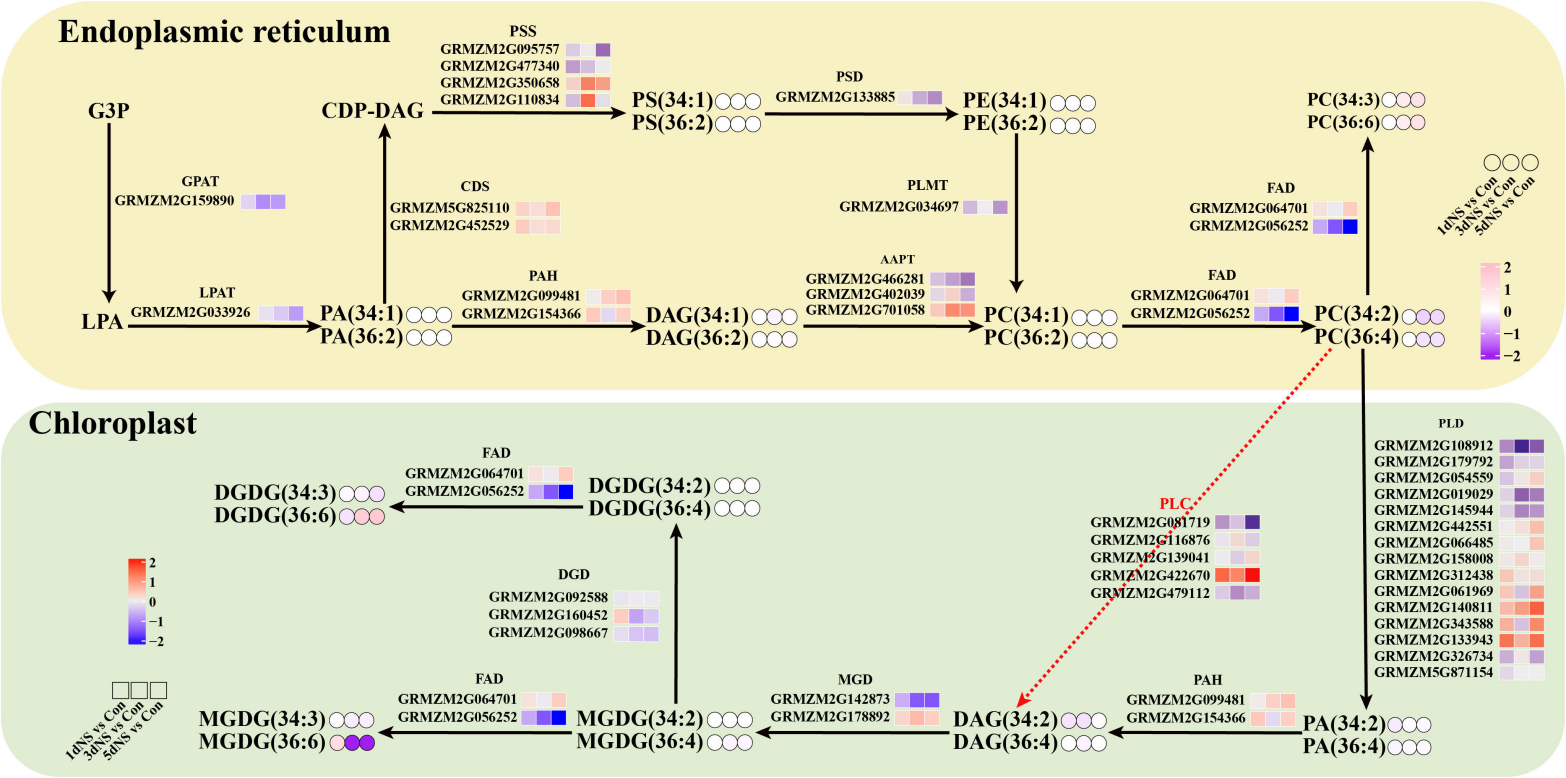
Gene-metabolite network diagrams demonstrate membrane lipid metabolism under inhibition. Differential expression levels of genes and different metabolite molecular species in major lipid metabolic pathways were presented in the form of heatmaps. Small squares represent heat maps of DEGs and small circles represent changes in lipid content.

In green tissues of plants, glycolipids (MGDG and DGDG) occupy a large portion of the body, and chloroplasts are the main site of glycolipid synthesis. A large part of the precursor DAG for glycolipid synthesis in chloroplasts comes from the degradation of phospholipids catalyzed by phospholipases. Phospholipases PLC and PLD are the major enzymes that catalyze the degradation of phospholipids, and the products are DAG and PA, respectively. Previous studies have shown that phospholipases have a preference for specific phospholipid species, such as 36:4 PC and 34:2 PC. As can be seen on **Figure 7**, when phospholipase C was inhibited, the content of its direct products 36:4 DAG and 36:4 DAG was reduced to a certain extent. At the transcriptional level, unigenes encoding PLC and PLD were also affected by phospholipase C inhibition, with some significantly upregulated and some repressed. The changes of MGDG and DGDG, the main components of chloroplast membrane lipids, were also inconsistent. MGDG gradually decreased under NS treatment, while DGDG showed an increasing trend. Since DGDG is a bilayer lipid, its increase maybe beneficial to maintain the stability of photosynthetic membrane lipids.

## 4 Discussion

A variety of plant lipids, such as phospholipids and glycolipids, are the main components that make up biological membranes (Seth et al., 2024). Phospholipids can be degraded by phospholipases into diacylglycerol (DAG) and phosphatidic acid (PA) (Zhu et al., 2020). In plants, phospholipases are categorized into three groups, namely Phospholipase A (PLA), Phospholipase C (PLC), and Phospholipase D (PLD) (Hong et al., 2016; Takáč et al., 2019). In our previous study, we found that when maize seedlings were treated with the PLC inhibitor neomycin sulfate (NS, 100 mM), their growth was hindered and photosynthesis was significantly weakened, along with diminished carbon metabolism activity (Wei et al., 2022).Among them, phospholipase C is considered an important lipid hydrolase in plants and animals with profound effects on membrane lipid remodeling and intracellular signaling (Meldrum et al., 1991). In the present study, the total molar percentage of phospholipids showed an increasing trend when phospholipase C was inhibited by 100 mM neomycin sulphate (NS), and further analysis revealed that the increase in PC and PE accumulation, which was partially due to the blockage of the PC hydrolysis pathway.

**Figure 8:**
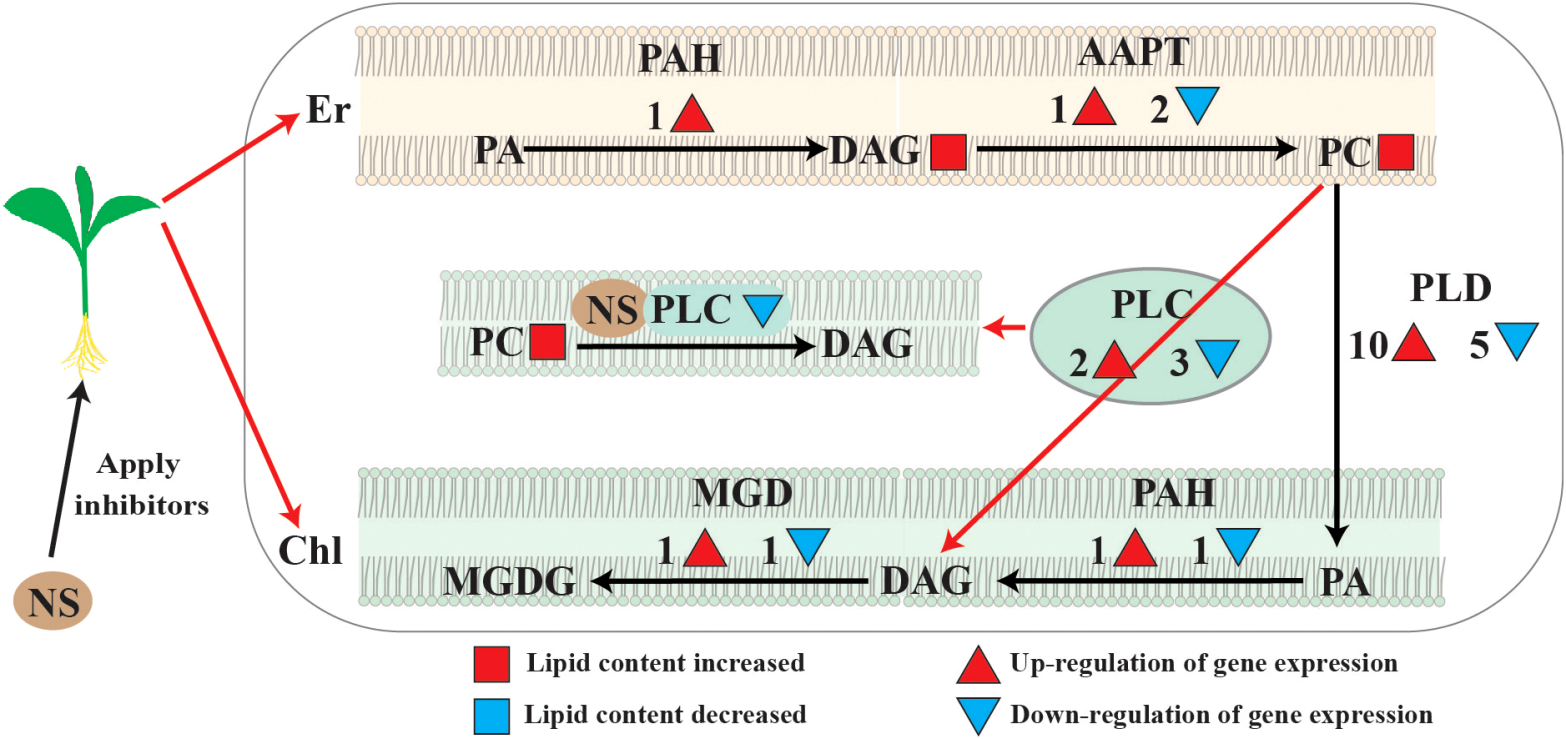
Major changes of lipid metabolism pathways under neomycin sulfate (NS) inhibition. Major changes in lipid metabolic pathways under neomycin sulfate (NS) inhibition. Red circles represent molar percentage content increase under inhibitory conditions. Numbers and red pos-itive triangles represent the number of up-regulated expressed genes, and dendrites and blue inverted triangles represent the number of down-regulated expressed genes.

It has been shown that PCs derived from the ER occupy a large proportion of the chloroplast outer membrane, and these PCs are also precursors for the synthesis of galactolipids in the chloroplast membrane (Yin et al., 2015; Botella et al., 2017). There are two main phospholipid hydrolysispathways in the chloroplast outer membrane (Xu et al., 2021; Ge et al., 2022), either through the PLD/PLPP pathway that produces PA and DAG in the chloroplasts, or through the PLC pathway that directly generates DAG in the chloroplasts(**Figure 8**) (Xu et al., 2021; Ge et al., 2022). In this study, most of the PLDs and PLPPs genes showed up-regulated expression; therefore, it is concluded that under conditions of phospholipase C inhibition, PC hydrolysis mainly occurs through the PLD pathway(**Figure 8**). When phospholipase C was inhibited, PA and DAG production through the PC degradation pathway was reduced, and more reliance was placed on the *de novo* synthesis pathway.

In plants, the membrane of chloroplasts is mainly composed of four distinct lipid classes, monogalactosyldiacylglycerol, digalactosyldiacylglycerol, sulfoquinuclidylglycerol, and phosphatidylglycerol (Kobayashi, 2016; Hölzl and Dörmann, 2019). Among them, MGDG and DGDG together account for about 80% of chloroplast lipids (Li and Yu, 2018). MGDG and DGDG are very important for plant photosynthesis, and a decrease in the MGDG/DGDG ratio is beneficial for maintaining chloroplast morphology (Ge et al., 2022). In this study, MGDG decreased and DGDG increased, leading to a decrease in the MGDG/DGDG ratio(**Figure 7**), which suggested that MGDG was converted to DGDG in order to maintain the stability of the chloroplast membrane (Xu et al., 2021; Ge et al., 2022; Wei et al., 2022).

In summary, when phospholipase C is inhibited, the DAG production *via* PLC hydrolysis is blocked, resulting in the accumulation of PA, DAG and PC. At the same time, the PLD/PAH pathway was activated in chloroplasts, and the PC accumulated in the endoplasmic reticulum were converted into PAs in chloroplasts through the PLD pathway and converted into DAG in chloroplasts through the PAH pathway. Therefore, the metabolic equilibrium of PC was disrupted and the membrane remodeling was impaired, which may affect the integrity and stability of both plasma membranes and chloroplast membranes

## Data Availability

The datasets presented in this study can be found in online repositories. The names of the repository/repositories and accession number(s) can be found in the article/**Supplementary Material**.

## Supplementary material

Supplementary Figure 1 Glycerol lipid correlation analysis under NS inhibition.

Supplementary Figure 2 Transcriptome analysis of maize seedling leaves under NS inhibition. A. Volcano plot of differentially expressed genes (DEGs); B. Upset diagram of DEGs

Supplementary Table 1 Lipid related gene expression data on Glycerophospholipid metabolism and Glycerolipid metabolism in leaves of maize seedlings under inhibited conditions.
