# Supplementary figures and images for: Effects of phospholipase C inhibition on the regulation of membrane lipid metabolism in maize leaves
Source: Front Plant Sci. 2025 Apr 1;16:1547477. doi: 10.3389/fpls.2025.1547477 (PMC11996868; doi:10.3389/fpls.2025.1547477)

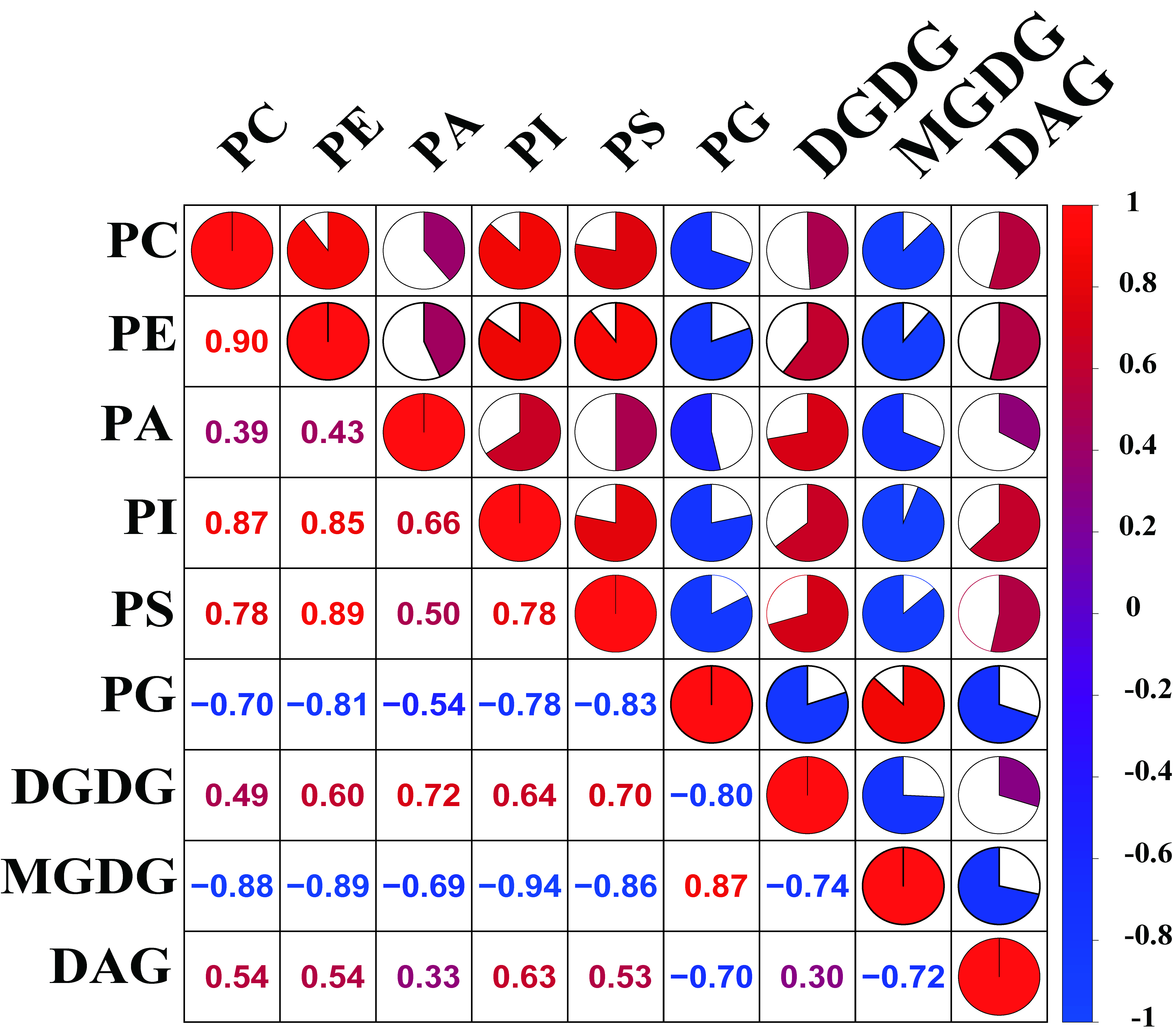

Supplement: Supplementary Figure 1 — Glycerol lipid correlation analysis under NS inhibition. [file Image1.jpeg]

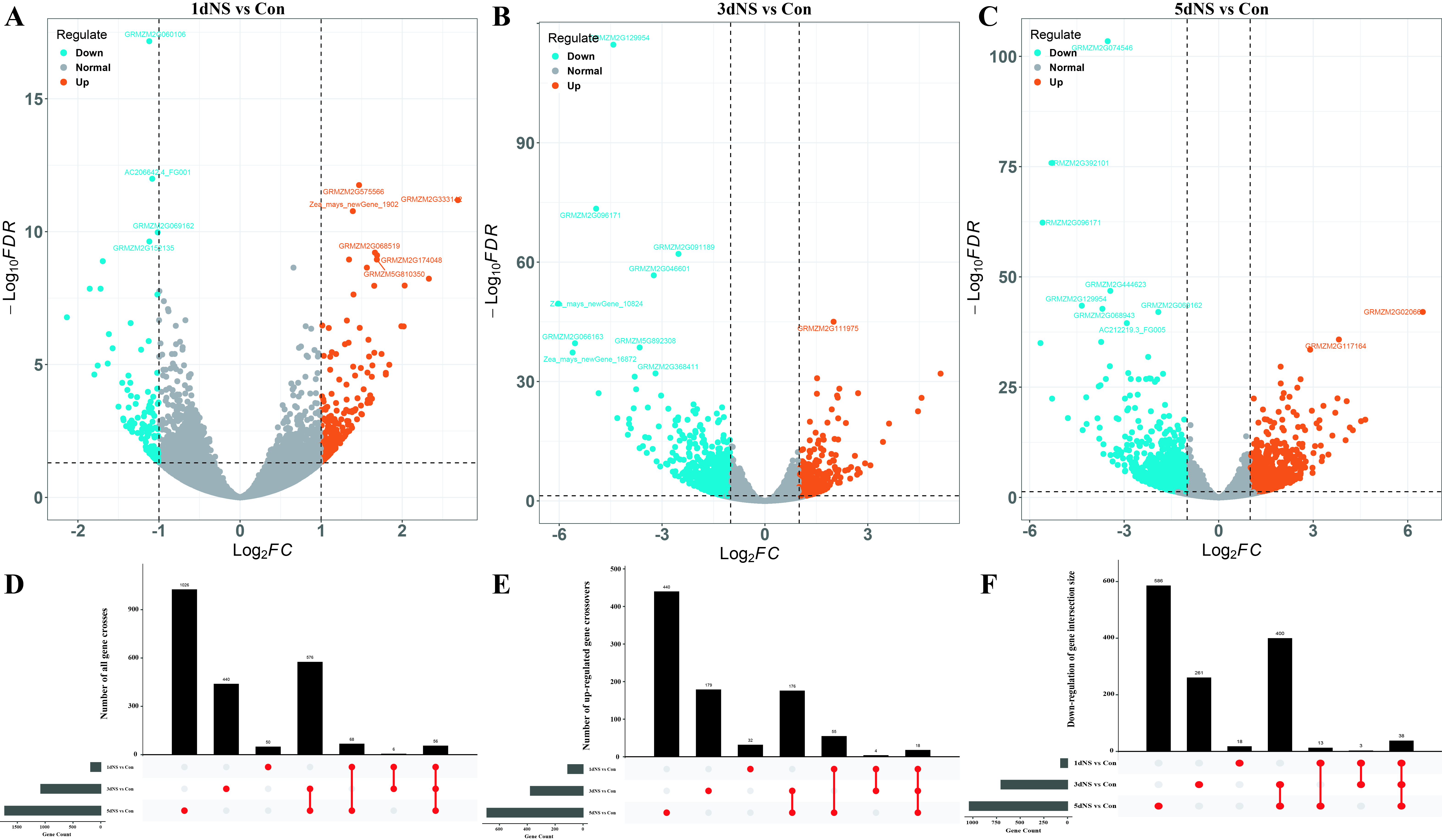

Supplement: Supplementary Figure 2 — Transcriptome analysis of maize seedling leaves under NS inhibition. A. Volcano plot of differentially expressed genes (DEGs); B. Upset diagram of DEGs [file Image2.jpeg]
